# Preventing Sexual Violence Against Adolescent Girls: Psychometric Validation of the EDR-ESIA Screening Instrument for Early Detection of Exploitation Risk

**DOI:** 10.3390/bs16050831

**Published:** 2026-05-21

**Authors:** Beatriz Benavente, Paola Bully, Lluís Ballester

**Affiliations:** 1 Department of Pedagogy and Specific Didactics, Faculty of Education, University of Balearic Islands, 07122 Palma de Mallorca, Spain; lluis.ballester@uib.es; 2 Paola Bully Methodological and Statistical Consultancy, 48071 Bilbao, Spain; paola.bulli@ehu.eus; 3 Department of Developmental and Educational Psychology, Faculty of Education, University of the Basque Country (UPV/EHU), 48940 Leioa, Spain

**Keywords:** child sexual exploitation, psychometric validation, risk assessment, gender difference, screening tool, prevention strategies

## Abstract

Sexual violence against women frequently originates during adolescence, when structural inequalities and gendered power dynamics heighten vulnerability, making early identification of risk factors essential to prevent trajectories leading to sexual exploitation. This study presents the psychometric validation of the EDR-ESIA, a screening instrument designed to detect vulnerability to Child Sexual Exploitation (CSE) in healthcare, education, and social care settings, with particular relevance for prevention strategies targeting adolescent girls. The sample comprised 199 adolescents aged 11–17 years (M = 15.23; SD = 1.59) residing in Spain (58.8% female, 40.2% male, 1.0% unspecified), assessed by trained professionals using case records and reports. The 88-item instrument underwent expert review and pilot testing prior to validation, and its internal structure was examined using Partial Least Squares Structural Equation Modeling (PLS-SEM). The results indicated that all subdimensions and higher-order constructs showed an adequate fit to the theoretical model, supporting the instrument’s validity. Female adolescents scored significantly higher than males on CSE target indicators, reflecting a medium-to-large gender difference in vulnerability levels. Overall, the EDR-ESIA constitutes an evidence-based instrument for the timely recognition of CSE vulnerability, supporting prevention, education, and intervention efforts aimed at reducing sexual violence against women from early developmental stages.

## 1. Introduction

Child sexual exploitation (CSE) happens when an individual or group takes advantage of a power imbalance to coerce, manipulate, or trick children or adolescents into performing a sexual activity in exchange for something the victim needs or wants, producing economic profit, privileges, or other advantages for the exploiter (ECPAT Interagency Working Group, 2016; Laird et al., 2023). There may be sexual exploitation even if the sexual activity seems to be consensual. CSE does not always involve physical contact; it may also occur with the use of technology (as is the case with pornography and Livestreaming Technology for Child Sexual Exploitation and Abuse, amongst others) (Finkelhor et al., 2023; Joleby et al., 2021; Lu et al., 2024).

CSE constitutes a serious violation of children’s rights, defined as “sexual abuse committed by the adult and remuneration in cash or kind to the child or a third person or persons” (Stockholm, 1996). This type of sexual victimisation, recognised as one of the most damaging public health problems (Engelmann et al., 2025; Greenbaum, 2020; World Health Organization, 2017), has gained prominence on the international and European political agenda. Spanish legislation, particularly Organic Law 8/2021, of 4 June, on the comprehensive protection of children and adolescents against violence (LOPIVI, 2021), explicitly addresses CSE as a type of violence that requires a comprehensive response.

The consequences of CSE have been well documented. The aftereffects of CSE tend to manifest themselves through symptoms at the behavioural, physical, psychological, social, and sexual level (Fredlund et al., 2013; Jackson, 2014; Pedersen & Hegna, 2003; Perry et al., 2022). The seriousness of these aftereffects is conditioned by a series of mediating variables such as a greater level of social stigma; difficulties in coping skills; a higher number, frequency, duration, and consequences of traumatic events; etc. The aftereffects of CSE have been linked to low self-esteem, a greater risk of re-victimisation, emotional dysregulation, and dysfunctional coping skills in adulthood, including substance use; they can be devastating and last a lifetime (Caffo et al., 2021; Benavente & Pereda, 2026). Evidence suggests that girls are more likely to develop internalizing symptoms (e.g., depression, anxiety), while boys may present externalizing behaviours (e.g., aggression, delinquency), which requires tailoring prevention and intervention strategies accordingly (Gauthier-Duchesne et al., 2017; Levkovich et al., 2025).

CSE, as a form of sexual violence against children and adolescents, presents particular and differential characteristics that define it as a multicausal and complex social problem that violates children’s right to protection and safety (Pereda et al., 2021; Benavente et al., 2024).

In the European context, the process of victim recruitment has been identified as differing from the methods prevalent in international networks of human smuggling and trafficking, where kidnapping, violence, and threats predominate. In Europe, however, it is based on the concept of exchange, as reported by Pereda et al. (2022). This exchange process arises from an imbalance of power that forces the victim to silence, generating a perception of responsibility and consensual participation in a sexual relationship (Laird et al., 2023). It is crucial to underline that not all cases involve victims being displaced to other areas or regions; instead, exploitation tends to take place in the same area where the victims and their exploiters—who do not always have links to organised crime—live (Greenbaum, 2018). This approach highlights the complexity and lack of uniformity in the modus operandi of child sexual exploitation in the European context. The prevalence of CSE in Europe stands at 1–2.5% for girls and 1–2.1% for boys, who have reported in different studies to have “traded with their sex” (Averdijk et al., 2019; Fredlund et al., 2013, 2018; Pedersen & Hegna, 2003). These percentages undergo a marked increase when referring to adolescents who live in protection institutions, reaching 17.4% in Spain (Pereda et al., 2023) and 24.4% in the United Kingdom (Lerpiniere et al., 2013). This underscores the notable vulnerability of this group, despite being scarcely studied.

From a perspective of gender, CSE has been evidenced to affect both boys and girls, defying previous stereotypes (Benavente et al., 2022b). Notwithstanding, recent studies suggest that the percentage of male victims could be underestimated, posing the possibility that boys might have greater difficulties in recognising themselves or being recognised as victims (ECPAT International, 2021; Josenhans et al., 2020; Madigan et al., 2018; Øien et al., 2025; UNICEF, 2020).

Sexual exploitation has also found new channels through Information and Communication Technologies (ICT), with the Internet as the space most used by perpetrators to recruit victims (Baird & Connolly, 2023; EUROPOL, 2020, 2025; Finkelhor et al., 2024). In fact, it can be used in a variety of ways by people with a sexual interest in children and adolescents; for instance, to make and distribute pornography, to recruit children and adolescents online, or to engage in or promote sexual relationships with children and adolescents (Ballester et al., 2020, 2022). Recently, the terms IBSA and IBSE have been proposed to refer to technology-facilitated image-based sexual abuse/exploitation, given the growing evidence of the prominent role that Internet technologies have in the growth of sexual abuse and of sexual exploitation aimed at the production of images and at the production of pornography (Ali et al., 2023; Finkelhor et al., 2023), by incorporating any technological novelty, such as, lately, Generative Artificial Intelligence or Livestreaming Technology and Online Child Sexual Exploitation and Abuse (Dorotic & Johnsen, 2023; Drejer et al., 2024). These diverse ways in which the pornographic industry is taking advantage of sexual images have increased the situations of sexual exploitation directed towards the production of exploitation images, as has been revealed in an evaluation of international studies (Weprotect, 2023; Pereda & Águila-Otero, 2026). Girls, in particular, are more frequently targeted through online grooming and image-based exploitation, reflecting broader patterns of sexual violence that disproportionately affect women and adolescent girls (de Santisteban & Gámez-Guadix, 2018; Mármol et al., 2025).

Precise knowledge of this phenomenon, including its indicators and risk factors, is crucial in order to prevent, identify, and effectively tackle CSE (Barnert et al., 2022; Benavente & Pereda, 2026). Further, the training of professionals is fundamental for early detection and adequate intervention, reducing the risk of secondary victimisation (Brady, 2018; Walsh et al., 2022). In this line, the effective fight against CSE requires not only awareness of the issue, but also the necessary willingness and knowledge to act in an effective way.

Although it is true that no child or adolescent is exempt from being a victim of CSE, certain experiences, situations, and contexts in the life of minors are linked to an increase in the risk of suffering this form of exploitation (Barnert et al., 2017; Pereda et al., 2025). It is essential to highlight the fact that there is no one single factor that can explain the risk of a minor’s involvement in CSE. Hence, it is imperative to understand the diverse factors that could have contributed to the minor becoming involved in this situation (Laird et al., 2023).

The importance of having effective tools and instruments for the detection of risk by professionals becomes evident in this context (Greenbaum, 2020; Weston & Mythen, 2023). These tools must be sensitive enough to identify the life experiences, situations, and contexts that could increase the risk of sexual exploitation. Likewise, the complexity of the factors that contribute to minors becoming involved in CSE must be addressed (Franchino-Olsen, 2021; Laird et al., 2023). In order to ensure the adequate implementation of these tools, it is essential to provide specialised training to professionals. This training not only guarantees the correct use of the instruments, but also boosts the in-depth understanding of the risk indicators, thereby strengthening the ability of professionals to intervene in an effective way and to protect vulnerable children and adolescents (Basson, 2017; Benavente et al., 2024).

In the last few years, various screening tools have been developed to help professionals in the identification of minors who are victims of or at risk of CSE; systematic reviews of instruments have even been conducted (Armstrong, 2017; Benavente et al., 2024; Interiano-Shiverdecker et al., 2022; Romero et al., 2021). Nonetheless, most of these tools were conceived in the United Kingdom and the United States of America, and the only one designed and validated in Spain is the Tool for the Detection of the Risk of Child Sexual Exploitation (EDR-ESIA) (Benavente et al., 2022a, 2024).

Basson et al. (2012) highlight that 75% of young individuals affected by commercial sexual exploitation face years of ongoing abuse before any intervention takes place, underscoring the importance of tools that facilitate decision-making for professionals. These tools enable them to assess the level of intervention required, based on whether the indicators suggest that the minor is at risk of being, or may already be, sexually exploited (Panlilio et al., 2022).

Despite their potential usefulness, it must be noted that very few of these tools have been subjected to a rigorous evaluation of their metric properties (Benavente et al., 2024). This lack of scientific evidence compromises the validity and reliability of their proper functioning in practice. In this sense, the lack of studies supporting the efficacy and accuracy of these tools raises questions as to their generalised application and highlights the urgent need to carry out rigorous evaluations to validate their usefulness and efficacy in the detection and evaluation of the risk of CSE, in a range of contexts and populations (Benavente et al., 2024). Recent studies, such as the one by Lu et al. (2024), continue to emphasize the need to improve metrics for conducting prevalence studies using well-established instruments with an operational definition. This enhancement would contribute to greater clarity in establishing consistent metrics. Given the documented gender differences in vulnerability, instruments such as the EDR-ESIA are crucial for equipping professionals with tools that can capture both common and gender-specific risk indicators of sexual exploitation.

In order to facilitate early detection and evaluation of CSE and how to approach it in the early stages of detection, it is key to provide professionals with reliable and valid instruments that will enable the detection of the factors associated with CSE. For this purpose, the main aim of this study was to analyse the psychometric properties of the EDR-ESIA, the previously designed screening instrument, which is considered the most comprehensive tool for detecting child and adolescent sexual exploitation in the Spanish population. (Benavente et al., 2022a, 2024). This instrument is based on a victim-centered approach, as proposed by Dixon (2024), but also includes others who may become victims (potential victims), following a universal prevention approach.

The primary hypothesis for the validation of the EDR-ESIA tool can be summarized into four key points:

Theoretical Model Fit: The internal structure of the EDR-ESIA—comprising target indicators, three levels of risk (significant, moderate, and other), and family/personal vulnerabilities—would show an adequate statistical fit to a theoretical model of Child Sexual Exploitation (CSE) risk.

Dimensional Contribution to Total Risk: That each subdimension (Target Indicators, Severe Risk, etc.) would make a meaningful and significant contribution to the higher-order construct of “CSE TOTAL RISK”. This was tested using Partial Least Squares Structural Equation Modeling (PLS-SEM) to see how well these factors predicted overall vulnerability.

Sensitivity to Gender Differences: Given that the tool integrates a gender perspective, the researchers hypothesized that it would identify statistically significant differences between sexes, specifically expecting female adolescents to show higher levels of vulnerability and higher scores in target indicators compared to males.

Psychometric Stability: The study operated on the premise that the formative indicators (situational observations) would remain reliable and valid across different groups (such as different ages and sexes), providing a stable framework for professionals to use in primary care settings.

## 2. Methods

### 2.1. Participants

The inclusion criteria defined for professionals were as follows: (1) Being a health or social care professional in either public or private institutions (doctors, nurses, social workers, psychologists, and other health care and social services professionals) or a member of the security forces (police officers and members of other security forces who work in the protection and defence of minors). (2) Having experience and direct contact with minors at risk of child sexual exploitation. This contact might be through direct interventions, prevention schemes, investigation of cases, or provision of support and rehabilitation services. (3) Having participated in training for the identification, prevention and intervention in cases of child sexual exploitation using the EDR-ESIA tool.

After meeting the inclusion criteria, professionals were invited to participate and provided informed consent prior to completing the questionnaire. Participants were informed that the data should be reported based on existing case records and that no identifying information about minors would be collected.

In order to ensure the integrity and validity of the results of the study (Shadish et al., 2002; Engelmann et al., 2025), the following exclusion criteria were designed: (1) professionals who had had no direct contact with minors in a situation of vulnerability or whose experience was limited to administrative or indirect functions with no direct intervention with boys, girls, and teenagers; (2) professionals with fewer than two years of experience in roles related to the care, protection, or safety of minors; and (3) participants who might have conflicts of interest that could compromise the objectivity of the data, such as financial ties or personal relationships with potential study subjects.

In order to ensure diversity and inclusiveness in the sample (Benavente et al., 2021), the selection criteria for minors at risk of sexual exploitation upon whom to report information for this study were: (1) Being between 11 and 17 years old; (2) Being under the supervision or protection of social services, foster homes, shelters, or child protection programmes; and (3) Being identified by authorities, social services, schools, or health professionals as a vulnerable population. To ensure the validity and ethics of the study, the following exclusion criteria were defined: (1) Not having been formally identified by professionals or the competent authorities as actually being at risk of sexual exploitation; (2) Being under 11 or over 17 years old; and (3) People whose participation in the study might place them at additional risk of victimisation or reprisals; situations in which participation in the study might compromise their personal safety or that of their family.

Out of the 325 times the tool was started through the online questionnaire, 73 were abandoned and 252 were completed, which represents a response rate of 77.54%. It was decided to exclude from the study the data of 51 individuals: 18 cases (7.1%) with missing responses to all 13 target indicators, two cases (0.01%) classified as factitious responses, and 33 cases (13.09%) where the participants’ age exceeded the established limit.

To assess the adequacy of the sample size, we conducted a sensitivity power analysis for the most complex endogenous construct in the model (five predictors). The results showed that, with N = 199, α = 0.05, and power = 0.80, the model was able to detect effects as small as f^2^ = 0.066. This indicates that the available sample size was sufficient to detect small-to-moderate effects. To evaluate the stability of the model, we conducted a bootstrap validation procedure with 5000 resamples across the multiply imputed datasets. The bootstrap results showed highly stable path estimates across resamples and imputations. All structural path coefficients remained significant, and the 95% confidence intervals consistently excluded zero. These findings support the robustness of the model despite the moderate sample size.

The 199 adolescents who finally made up the sample were between 11 and 17 years old (*M* = 15.23; *SD* = 1.59). Their sociodemographic characteristics can be observed in the following table (Table 1):

Of the sample explored, 133 (66.8%) had a dossier open in Social Services and 92 (46.2%) had a family dossier open in the same service. One hundred and seventy-eight tools (89.4%) were completed for boys and girls with a dossier open in child protection and 24 (12.1%) with a dossier open in juvenile justice. Regarding their home, 131 (66.4%) of the children and adolescents were in protective residential care, 44 (22.4%) lived in the family home, 11 (5.6%) in family foster care, 8 in juvenile justice residential care (3.9%), while for 5 (1.7%) this detail was unknown.

### 2.2. Instrument

The final version of the EDR-ESIA tool subjected to validation (Benavente et al., 2024) is made up of 88 items to be completed by a professional, with information gathered from the reports, medical history, and/or dossiers of the minor in four sections: (1) Identification of the minor and their family (22 items), (2) CSE target indicators (13 items), (3) Risk indicators subdivided, in turn, into: (a) Indicators of significant risk; (b) Indicators of moderate risk; (c) Other indicators of risk; with 12 items in each subcategory (up to 49 items in all the indicators); and (4) Vulnerabilities of the minor (17 items). The characteristics of the indicators considered are based on descriptive language; therefore, they leave very little margin for distorted evaluations. Technically, they are observations from professionals of situations that might be described; if the situation can be documented, the indicator always works in the same way in different informants.

To facilitate their completion, each of the risk indicator items is defined in the tool itself and indications are given as to how to score them according to the degree to which they are manifested. The score for each of these items is recorded as not present: 0; mild: 1; moderate: 2; serious: 3, with the following score considered as the final result: >9 points: established risk; 6–9 points: likely risk; 1–5 points: at professional discretion. In addition, risk is considered to be established whenever the first target indicator is scored differently from 0. The EDR-ESIA tool was designed for application in first-level detection in care settings (Benavente et al., 2022a, 2024). The EDR-ESIA is intended to be applied in primary care education, health, and social services.

### 2.3. Procedure

**Figure 1:**
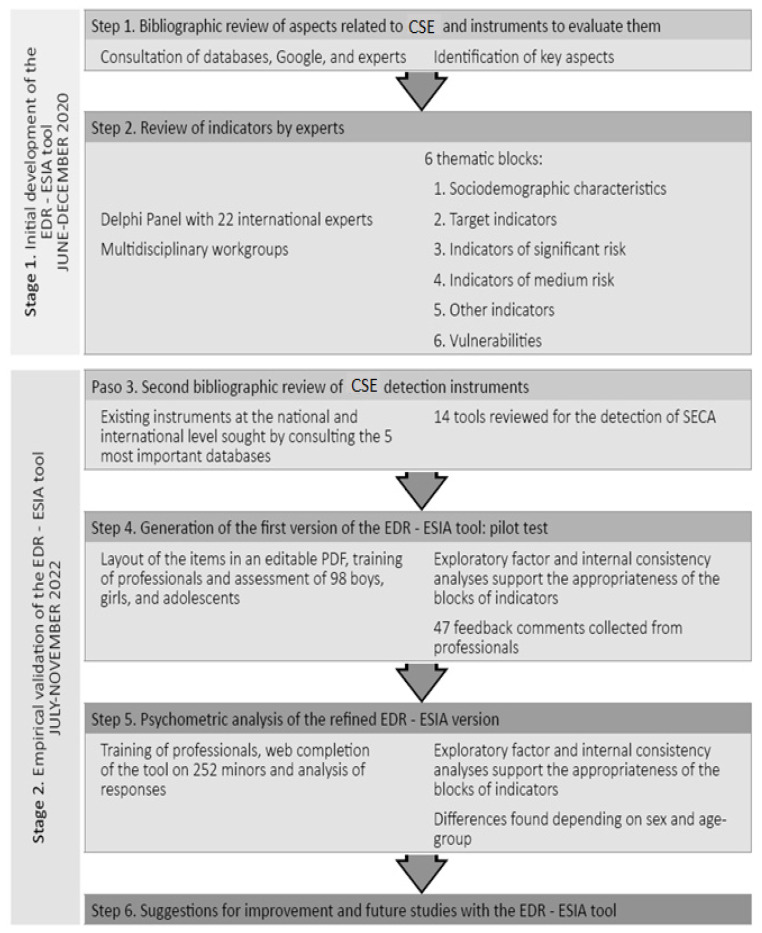
Flow diagram of the development process and analysis of metric properties. Source: Authors’ own elaboration. Note. SECA = Sexual Exploitation of Children and Adolescents.

The EDR-ESIA tool was developed in a 2-stage process, covering the six steps necessary for its development. Figure 1 serves as an outline of the work process followed.

The flow diagram (Figure 1) provides a comprehensive overview of the EDR-ESIA tool’s evolution, detailing a two-stage process composed of six specific steps:

Stage 1: Initial Development (June–December 2020):

Step 1: Conducted a bibliographic review of aspects related to Child Sexual Exploitation (CSE) and identified key evaluative indicators.

Step 2: Involved a review of indicators by a Delphi Panel of 22 international experts and multidisciplinary workgroups to establish six thematic blocks: sociodemographic characteristics, target indicators, and various levels of risk and vulnerability.

Stage 2: Empirical Validation (July–November 2022 onwards):

Step 3: Executed a second bibliographic review specifically targeting 14 existing national and international CSE detection instruments.

Step 4: Generated the first version of the tool in an editable PDF format, followed by professional training and a pilot test involving 98 minors, which gathered 47 feedback comments from users.

Step 5: Performed a psychometric analysis of the refined version, utilizing web completion for 252 minors (with 199 cases meeting the final inclusion criteria) and employing Partial Least Squares Structural Equation Modeling (PLS-SEM) to confirm the model’s fit.

Step 6: Outlined suggestions for improvements and future longitudinal studies.

The current validation study specifically focuses on the last two steps of this flow diagram, analyzing the metric properties and structural validity of the final refined instrument. This expanded description offers a holistic view of how the tool transitioned from a qualitative expert consensus (Stage 1) to a statistically validated screening resource (Stage 2).

### 2.4. Data Analysis

First of all, a preliminary analysis of the information collected was conducted in order to refine the data and determine the distribution of frequencies in each of the elements. For the evaluation of the theoretical model, Partial Least Squares Structural Equation Modelling (PLS-SEM) methodology was used, which is particularly suitable for models with formative constructs, since it assumes no correlation between indicators and allows for the analysis of the individual contribution of each indicator to the latent variable (Hair et al., 2022; Sarstedt et al., 2022). This approach is suitable when indicators represent different dimensions of the construct rather than reflecting a single underlying entity, which is consistent with the specification of the present study (Diamantopoulos & Winklhofer, 2001; Hair et al., 2022).

In the specified model, first- and second-order factors are considered, all of a formative nature. The first-order factors include specific indicators that capture different dimensions of risk and vulnerability. In particular, (1) Target Indicators (ID): composed of 13 indicators (ID1–ID13); (2) Severe Risk Indicators (IRS): Composed of 12 indicators (IRS1–IRS12); (3) Medium Risk Indicators (IRM): Made up of 12 indicators (IRM1–IRM12); (4) Other Risk Indicators (OIR): Contains 12 indicators (OIR1–OIR12); (5) Family vulnerability (VF): Composed of 10 indicators (VF1–VF10); and (6) Personal vulnerability (VP): Defined by 7 indicators (VP1–VP7).

Given that the first-order factors represent differentiated dimensions of risk and vulnerability, their indicators were modelled formatively. In turn, second-order relationships were established in the structure of the model: Global vulnerability (V): this is a second-order formative construct that is determined by its two first-order dimensions, VF and VP. Global risk (CSE TOTAL RISK): This is a second-order construct, predicted by the four risk factors (ID, IRS, IRM and OIR) and by global vulnerability (V).

Responses coded as “unknown” were treated as missing values rather than as the absence of risk. Because several indicators showed non-trivial proportions of missingness, missing data were handled using multiple imputation by chained equations (MICE) as the primary strategy. To assess robustness, the PLS-SEM was re-estimated using median imputation as a sensitivity analysis. The results were highly consistent across missing-data strategies. Differences between multiple imputation and median imputation were negligible for all structural paths (all absolute differences < 0.002) and for the explained variance of the endogenous constructs (absolute differences in R^2^ < 0.001). Measurement parameters were also broadly stable, with only minor variation in a small number of indicator weights and loadings. Overall, the substantive conclusions of the model were unchanged across imputation methods.

The weights of the indicators were also analysed to assess the contribution of each indicator to the formative construct and their statistical significance using the PLS algorithm with bootstrap resampling of 5000 iterations (Chin, 1998). Since the constructs are formative, internal reliability was not assessed using Cronbach’s Alpha or Composite Reliability, as these indicators assume homogeneity across items, which is not applicable to formative models (Diamantopoulos & Siguaw, 2006; Hair et al., 2022). Instead, collinearity between indicators was examined using the Variance Inflation Factor (VIF), ensuring that no indicator exceeded a threshold of 5, which would indicate redundancy problems (Diamantopoulos & Winklhofer, 2001; Hair et al., 2022).

To assess the overall quality of the PLS-SEM model, the coefficient of determination (R2), the Global Goodness of Fit (GOF) index, the effect sizes (f2) of each sub-dimension and the Stone–Geisser indices (Q″) were calculated (Hair et al., 2022).

Finally, the scores for each first-order factor were calculated, and differences were estimated based on sex and age group using non-parametric tests (Mann–Whitney U test). To complement the significance tests, effect sizes were calculated using Cohen’s d to assess the magnitude of the observed differences. This analysis aimed to identify potential variations in risk perception and vulnerability levels across demographic segments, providing a deeper understanding of how these factors may influence the overall assessment of ESIA risk.

All analyses were performed using the R statistical programme (R x64 version 3.5.0).

## 3. Results

### 3.1. Dimensionality

The formative measurement model showed generally consistent outer weights, supporting the adequacy of the construct specification. Although some indicators displayed relatively low weights, their relevance was assessed considering both outer weights and outer loadings in line with PLS-SEM recommendations (Hair et al., 2022). Indicators with low weights but meaningful loadings were retained, as a sensitivity analysis excluding them did not alter the model structure or substantive conclusions, and they remain theoretically and clinically relevant according to expert consultation during instrument development.

To assess potential collinearity among formative indicators, variance inflation factors (VIF) were calculated. As shown in Table 2 and Table 3, all VIF values were below conventional thresholds, indicating no problematic multicollinearity.

Vulnerabilities were a two-dimensional scale with appropriate and meaningful weights for each of the indicators as shown in Table 3.

**Figure 2:**
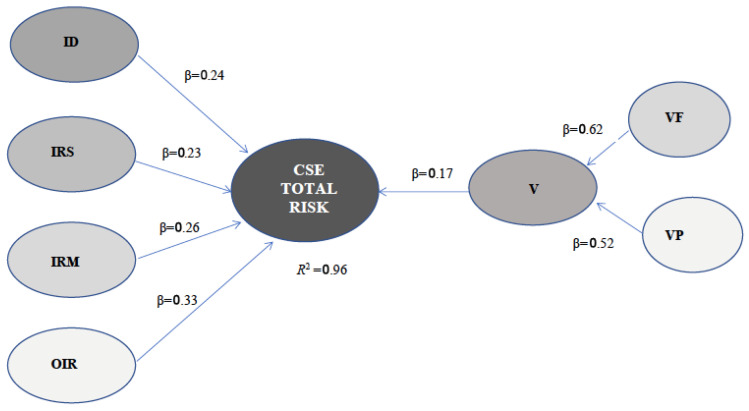
Estimated PLS-SEM structural model of CSE total risk. Source: Authors’ own elaboration. Note. Standardized path coefficients (β) are reported on the arrows. The coefficient of determination (R^2^) indicates the proportion of variance explained in the endogenous construct. Abbreviations: CSE = Child Sexual Exploitation; ID = Target Indicators; IRS = Severe Risk Indicators; IRM = Medium Risk Indicators; OIR = Other Risk Indicators; V = Vulnerabilities; VF = Family vulnerability; VP = Personal vulnerability.

Furthermore, the model meets the criteria for both discriminant and convergent validity. The discriminant validity assessment confirms that each construct is distinct from the others, as indicated by higher loadings on their respective latent variables compared to cross-loadings on other constructs. Furthermore, the coefficients of correlations between latent variables show that the risk constructs are highly correlated with each other (ID-IRS = 0.70, ID-IRM = 0.73, IRS-IRM = 0.73, OIR-IRM = 0.76), but maintain sufficient separation with respect to vulnerability (VF-VP = 0.50) (Figure 2). 

Convergent validity is also supported, as the indicators sufficiently explain the variance of their corresponding constructs. These findings suggest that the measurement model effectively captures the underlying theoretical structure.

The bootstrap analysis further confirms the stability of the estimated coefficients. By resampling the data and generating confidence intervals, the bootstrap procedure provides evidence that the estimated relationships are not highly sensitive to sampling variability. The absence of confidence intervals containing zero supports the significance of the estimated paths, reinforcing the reliability of the model’s structural relationships.

To further examine the psychometric performance of the proposed questionnaire, the structural model was assessed using the coefficient of determination (R^2^), effect sizes (f^2^), Stone–Geisser predictive relevance (Q^2^), and the global goodness-of-fit (GOF) index.

The results showed very high explanatory power for the endogenous constructs included in the measurement model. Specifically, the vulnerability construct (V) explained 97.6% of the variance (R^2^ = 0.97), while the higher-order risk construct (CSE TOTAL RISK) explained 98.2% of the variance (R^2^ = 0.98). As expected in the specification of the structural model, exogenous constructs (ID, IRS, IRM, OIR, V_Fam, and V_Pers) showed R^2^ values equal to zero.

Effect size analysis (f^2^) indicated that all sub-dimensions made substantial contributions to the explanatory capacity of the instrument. Within the vulnerability construct, both family vulnerability (V_Fam → V: f^2^ = 12.20) and personal vulnerability (V_Pers → V: f^2^ = 8.30) exhibited very large effects. Likewise, all dimensions contributing to the higher-order risk construct displayed large effect sizes, including OIR (f^2^ = 2.34), vulnerability (f^2^ = 1.42), ID (f^2^ = 1.28), IRS (f^2^ = 1.17), and IRM (f^2^ = 1.07). These results indicate that each dimension contributes meaningfully to explaining the overall construct measured by the instrument.

Predictive relevance was assessed using the Stone–Geisser Q^2^ statistic obtained through blindfolding. The results indicated strong predictive relevance for the endogenous constructs, with Q^2^ values of 0.97 for vulnerability and 0.98 for overall risk, both well above zero. According to established guidelines, Q^2^ values greater than zero indicate that the model has predictive relevance for the corresponding endogenous constructs.

At the global level, the model yielded a GOF index of 0.57, indicating a satisfactory overall fit between the measurement and structural components of the model.

It is important to note that the relatively high R^2^ and Q^2^ values should be interpreted in light of the hierarchical component model adopted in this study, in which higher-order constructs are formed by theoretically related sub-dimensions derived from the same measurement instrument. In such models, strong explanatory relationships between lower-order dimensions and higher-order constructs are expected, reflecting the conceptual coherence of the measurement structure rather than model overfitting.

### 3.2. Differences According to Sex and Age Group

As expected, the group of females obtained significantly higher mean scores than males in the target indicators, indicators of moderate risk, other indicators, and vulnerabilities to CSE (see Table 4).

Differences by sex showed that females reported higher scores than males in several domains. Moderate effect sizes were observed for CSE target indicators (d = 0.63), indicators of moderate risk of CSE (d = 0.74), other indicators of risk of CSE (d = 0.52), and vulnerabilities (d = 0.50). In contrast, the difference in indicators of significant risk of CSE was negligible (d = −0.02).

Regarding age group, adolescents aged 14–17 reported higher levels of CSE-related indicators than those aged 11–13 in several domains. Effect sizes were moderate for CSE target indicators (d = 0.54) and indicators of significant risk of CSE (d = 0.58), and large for indicators of moderate risk of CSE (d = 1.01). Differences in other indicators of risk of CSE (d = 0.11) and vulnerabilities (d = 0.03) were small or negligible (see Table 4).

### 3.3. Cut-Off Points

**Figure 3:**
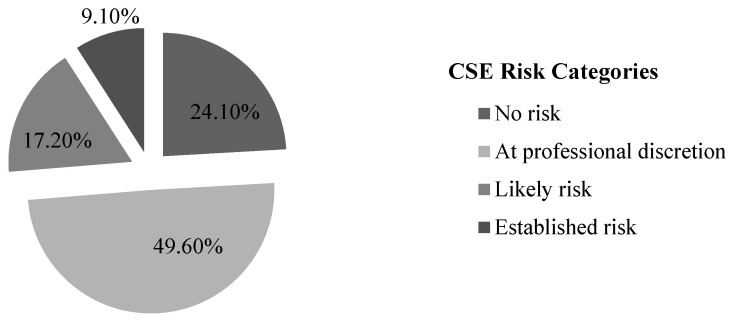
Frequency and percentage of participants in each level of risk of CSE. Source: Authors’ own elaboration.

This total score in target indicators can be divided into three sections or categories: (1) more than 9 points = established risk; (2) from 6 to 9 points = likely risk; and (3) from 1 to 5 points = at professional discretion. Based on this classification, 40 of those assessed (17.2%) were at likely risk of CSE, and 21 (9.1%) at established risk, that is, over a quarter of the sample (Figure 3).

## 4. Discussion

One of the greatest strengths of this tool is the use of a participatory approach for its development (Benavente et al., 2022a, 2024), by obtaining evidence of the validity of its content based on scientific literature and in a consensual and collaborative way with experts and professional users in a range of fields. Importantly, the instrument allows not only for a general assessment of CSE risk but also for a gender-differentiated study, making it possible to highlight the particular vulnerability of girls and adolescent women. Previous research shows that they are more frequently victims of exploitation and tend to develop internalizing consequences (e.g., depression, anxiety, PTSD), while boys are comparatively more likely to present externalizing symptoms (e.g., aggression, delinquency) (Euser et al., 2013; Lewis et al., 2016; Øien et al., 2025). The instrument also includes important information for analyzing the specific situations of boys who suffer sexual exploitation, in accordance with the need identified by Connella et al. (2023).

The use of PLS-SEM allowed the hierarchical structure of the formative constructs in the ESIA risk measure to be analysed, providing robust estimates and allowing the identification of factors contributing to the overall risk. The results indicate that all the dimensions of the EDR-ESIA questionnaire, from the metric point of view, have an adequate overall fit to the theoretical starting models. None of the items, except for the item “Minor is a sexual aggressor”, reveals local goodness-of-fit problems, with all saturating above 0.4 in their respective dimensions (mostly above 0.60). In the case of the “Minor is a sexual aggressor” item, this works differently from the rest, with a very low loading factor, due to its low frequency. Nonetheless, as it is an indicator of having suffered sexual violence, it is kept on the list. In terms of reliability, the internal consistency can be said to be very good in all dimensions. Therefore, it can be stated that the scores that will be obtained with the final version of the EDR-ESIA tool have the guarantees of validity and reliability demanded by the international standards for test creation and adaptation.

The use of a common tool is important for all professionals who work with minors because it enables the detection of children and adolescents at risk of or actually in a situation of sexual exploitation, by providing a common framework of reference that would act as a support for clinical judgement and subsequent measures to be taken. This is particularly relevant for girls and adolescent women, who are frequently targeted through online grooming or solicitations by adults—a phenomenon shown to be highly prevalent in longitudinal studies (Ortega-Barón et al., 2022)—and whose vulnerability is further confirmed by national data indicating gender-specific patterns of CSE in Spain (Pereda et al., 2025). In the short term, the functionalities of the EDR-ESIA tool are to: (1) enable the diagnosis or detection of minors at risk of CSE, by gathering information from completion of the tool in one of its different formats (paper- or web-based questionnaire); (2) establish priorities for in-depth exploration, by means of an interview, when indications are inconclusive; (3) compile and unify the main CSE risk indicators so that all the services responsible for care in childhood and adolescence have this information available; and (4) provide information to the professionals responsible for the care of minors regarding their lifestyles and patterns of greatest risk of CSE for the purpose of supporting them, motivating them, and advising them on how to approach the situation and encourage healthier lifestyles. In the medium term, it will serve to: (5) identify subpopulations at high risk of CSE, based on the data stored with the use of the tool; (6) be able to evaluate the prevalence of the risk of CSE at the population level, by using it in several settings such as schools, healthcare centres, and community services, amongst others; (7) characterise this population and plan the need for specific services; (8) contribute to monitoring accompaniment processes by means of changes in level of risk throughout the socio-educational work carried out; and (9) the EDR-ESIA tool on many occasions involves intersectoral collaboration between organisations that work at the community, welfare, and legal level, which promotes the development of synergies and new professional roles that can lead to programmes of joint participation between several organisations with a common purpose.

### Limitations and Future Research Directions

While the tool is built on a rigorous evidence-based development process, its current validation presents several limitations related to the characteristics of the sample and the predictive scope of the study, which are well documented in the methodological literature on risk assessment and validation studies (Shadish et al., 2002) and continue to be highlighted in more recent research on instruments for identifying child sexual exploitation (Benavente et al., 2024).

The evidence-based development of the instrument was supported by a multi-stage process that included a systematic review of the international literature, a Delphi panel with 22 international experts, a review by 36 active professionals in the field, and a methodological review conducted by university researchers. This process ensured that the indicators were grounded in established scientific knowledge prior to the empirical validation phase. However, this does not eliminate the limitations associated with the design of the validation study.

First, the instrument is a screening (not diagnostic) tool designed for early detection in normalized settings such as schools and health centers. Its primary aim is to provide a common framework of reference that can help non-specialized professionals identify alert signals. Therefore, it should be understood as a promising starting point for prevention strategies rather than definitive evidence of real-world impact, which is consistent with how similar tools in this field have been conceptualized in previous research (Benavente et al., 2024).

Second, previous studies have noted that many instruments addressing child sexual exploitation have been developed and validated in selective prevention contexts involving populations already identified as being at higher risk (Cockbain et al., 2017; Benavente et al., 2024). Consistent with this, in the most recent validation, the sample (N = 199) consisted of adolescents already under supervision or identified as vulnerable, which limits the generalizability of the findings to the general adolescent population.

Third, the cross-sectional nature of the current validation constitutes another important limitation. As widely recognized in evaluation research, cross-sectional designs restrict the ability to establish predictive validity and causal inferences over time (Shadish et al., 2002). Longitudinal research will therefore be necessary to determine the extent to which these indicators can prospectively predict situations of sexual exploitation, an issue that has also been highlighted in recent reviews of the field (Benavente et al., 2024).

Additional methodological limitations should also be considered. Although several indicators contained substantial proportions of “unknown” responses, the sensitivity analyses suggest that the main conclusions are robust to the choice of missing-data treatment. Nevertheless, this aspect should be interpreted with caution and further studies with more complete data collection procedures would strengthen the evidence.

A further limitation relates to reliability assessment. Because ratings were conducted retrospectively using information extracted from case files, it was not possible to systematically evaluate test–retest or inter-rater reliability, as multiple independent ratings were not available for all cases. While this approach is common in initial validation studies based on retrospective data, future research should address this limitation by incorporating repeated assessments and independent raters to further examine the temporal stability and inter-rater reliability of the instrument.

Finally, it is important to emphasize that a high score on the instrument does not provide certainty that exploitation is occurring, nor does a low score guarantee that a minor is free of risk. The tool is intended to support, rather than replace, professional clinical judgment and should be followed by a specialized assessment when risk is identified.

Future research should address the current limitations of the study while also adapting to the evolving nature of child sexual exploitation (CSE). First, further work is needed to strengthen the psychometric evaluation of the instrument. In particular, future studies should move beyond retrospective case-file analysis and incorporate repeated assessments and independent raters. This would allow for a systematic evaluation of test–retest and inter-rater reliability, which could not be fully assessed in the present study.

Second, longitudinal research is necessary to examine the predictive validity of the EDR-ESIA indicators over time. Such studies would help determine whether early identification of risk effectively anticipates trajectories into exploitation and supports preventive intervention.

Third, validation should be extended to broader and more diverse samples. Future studies could include adolescents from the general population and involve professionals from different regions to assess the applicability of the tool across contexts. Expanding validation to other Southern European settings may also be valuable in exploring potential cultural and linguistic adaptations of the instrument.

In addition, research should continue to refine the indicators associated with technology-facilitated sexual exploitation. There is a recognized need to improve the detection and assessment of Image-Based Sexual Exploitation (IBSE) in order to better evaluate risks associated with the online distribution of sexual images, following the recommendations developed by Minihan et al. (2024), Seto et al. (2024), and van der Spuy et al. (2024). Similarly, it is essential to improve the detection and prevention of situations involving child sexual exploitation material (CSEM), particularly when it is self-produced by minors under coercion, as suggested by Bloxsom et al. (2024).

Future research should also incorporate the perspectives and lived experiences of victims to better understand the pathways and contextual factors associated with exploitation. This may contribute to improving both the conceptualization of risk indicators and the design of preventive strategies.

Finally, special attention should be given to specific vulnerabilities that may be underrepresented in current screening tools, including adolescents from the LGTBIQ+ community or those with prior experiences of sexual victimization. These considerations are particularly relevant in the case of girls, who are disproportionately affected by online grooming and image-based coercion, reinforcing the need for preventive actions and interventions that integrate a gender perspective (Ortega-Barón et al., 2022; Fry et al., 2025). Altogether, these efforts may contribute to the development of a more comprehensive framework for addressing the increasing complexity of online child sexual exploitation.

## 5. Conclusions

This study aimed to validate the EDR-ESIA as a screening tool for the early detection of risk indicators associated with child sexual exploitation (CSE). The main hypothesis proposed that the internal structure of the instrument—comprising target indicators, different levels of risk, and contextual vulnerabilities—would show an adequate fit within a theoretical model of CSE risk and contribute meaningfully to the overall construct of total risk.

The results provide empirical support for the proposed hypotheses within the scope of the study design. The analyses indicated that the proposed theoretical model demonstrated an adequate statistical fit and that the different dimensions of the instrument contributed significantly to the higher-order construct of CSE total risk. In addition, the findings suggest that the indicators included in the EDR-ESIA are relevant for identifying patterns of vulnerability associated with exploitation.

Consistent with the study expectations, the instrument also showed sensitivity to gender differences, with female adolescents presenting higher levels of vulnerability and higher scores in several indicators, which aligns with existing research highlighting gendered patterns of risk in CSE. Furthermore, the formative structure of the indicators showed adequate psychometric behavior within the PLS-SEM model, supporting their usefulness as part of a screening framework.

Overall, the findings suggest that the EDR-ESIA may represent a promising screening tool to support early detection efforts in professional contexts such as education, health, and social services. At the same time, the results should be interpreted considering the limitations of the study, particularly the characteristics of the sample and the cross-sectional design. Continued research will be necessary to further consolidate the predictive validity and applicability of the instrument in broader populations and contexts.

## Figures and Tables

**Table 1:** Sociodemographic characteristics of the participants in the data collection with the refined version of the EDR-ESIA tool.

Characteristics	n	%
Sex		
Female	117	58.8
Male	80	40.2
Unknown	2	1.0
Age group		
11–13 years	34	17.1
14–17 years	160	80.4
Unknown	5	2.5
Autonomous community		
Balearic Islands	6	3.0
Galicia	8	4.0
Basque Country	86	43.2
Cantabria	98	49.2
Unknown	1	0.5
Legal situation		
Spanish nationality	138	69.3
Regularised/or	39	19.6
Migrant	11	5.5
Unaccompanied migrant/or	5	2.5
Unknown	6	3.0
LGTBI collective		
Yes	16	8.0
No	151	75.9
Unknown	32	16.1
Minority ethnicity		
Yes	46	23.1
No	146	73.4
Unknown	7	3.5
Educational level		
Primary	59	29.6
Secondary	103	51.8
Baccalaureate	4	2.0
VET	23	11.6
Dropped out	2	1.0
Unknown	8	4.0
Special Educational Learning Needs		
Yes	52	26.1
No	139	68.9
Unknown	8	4.0
Proficiency in the official language		
Yes	167	83.9
No	19	9.5
Unknown	13	6.5

**Table 2:** Percentage of participants in the different response options and factor loadings to CSE indicators.

	Not Present	Mild	Moderate	Serious	Unknown		
	%	%	%	%	%	λ	VIF
CSE Target Indicator (ID)							
ID1. Receives goods in exchange for sex	72.8	2.2	1.7	3.0	15.1	0.62	2.49
ID2. Recruiter of other minors for sexual exploitation	78.9	1.3	0.0	0.4	13.8	0.30	2.49
ID3. Unjustified possession of money, jewellery, mobiles, or other objects of value	68.5	15.5	7.3	1.3	3.0	0.58	2.49
ID4. Sexually active 13-year-old minor	73.7	5.2	3.0	2.2	7.8	0.34	2.49
ID5. Risky sexual relations	35.8	15.9	9.9	11.2	23.7	0.75	2.49
ID6. Involved in online sexual activity	53.9	10.8	3.0	3.4	27.7	0.44	2.48
ID7. Recurring sexually transmitted infections	75.4	6.5	0.4	0	12.1	0.52	2.47
ID8. Acquaintances and/or friends connected to sexual exploitation	57.3	6.0	1.7	3.0	27.6	0.47	2.47
ID9. Relations with people and places close to prostitution	61.2	3.9	1.7	2.2	25.4	0.54	2.45
ID10. Friends and/or partners older than the minor (5years of difference)	50.4	9.5	11.6	7.8	15.9	0.67	2.40
ID11. Online relationships and/or meetings with strangers	50.4	7.3	5.6	4.7	27.6	0.47	2.23
ID12. Abuse/dependence on alcohol and/or other drugs	47.8	22.8	11.2	10.3	3.4	0.68	1.98
ID13. Recurring physical injuries of unknown origin	77.6	4.3	2.6	0.9	8.6	0.44	1.20
Indicator of Significant Risk (IRS)							2.09
IRS1. Hiding information	23.7	31.9	25.0	12.1	5.2	0.77	1.58
IRS2. Providing inconsistent stories	44.8	22.8	15.5	8.2	4.7	0.74	1.57
IRS3. Running away from home	65.1	13.4	7.3	5.6	3.4	0.67	1.57
IRS4. Truancy	69.4	7.8	6.9	8.6	2.6	0.59	1.57
IRS5. Addiction to online or in-person gambling	76.7	2.6	2.2	2.6	10.8	0.16	1.56
IRS6. Verbalization of knowledge of the “Sugar Daddy” or “Sugar Dating” offer and the intention of making money from it	67.7	14.4	1.7	0.9	11.2	0.37	1.56
IRS7. Involvement in dysfunctional gangs/groups of friends	64.2	12.5	6.5	6.5	5.2	0.59	1.56
IRS8. Commission of crimes	69.0	10.3	5.6	3.9	6.0	0.44	1.55
IRS9. Weapon possession	84.5	1.7	0.0	2.2	4.7	0.14	1.52
IRS10. Tattoos that would indicate belonging to a gang or gang ownership	90.9	0.4	0.0	0.0	3.0	0.39	1.51
IRS11. Minor involved in drug trafficking	76.7	5.2	1.3	0.9	10.3	0.44	1.48
IRS12. On-line grooming	66.8	0.9	0.9	0.4	25.4	0.47	2.41
Indicators of Moderate Risk (IMR)							2.41
IRM1. Arriving home late	68.2	17.2	7.8	9.1	4.3	0.62	2.41
IRM2. Self-harm	72.4	11.2	5.6	2.2	3.9	0.55	2.40
IRM3. Suicidal ideas or attempts	69.4	11.6	5.2	1.3	5.6	0.35	2.39
IRM4. Occasional alcohol consumption	43.5	33.6	8.2	6.0	4.7	0.63	2.38
IRM5. Occasional drug consumption	56.9	14.2	6.9	9.5	7.8	0.66	2.37
IRM6. Sentimental relationships with a high level of emotional dependence	44.8	12.1	16.4	9.9	13.8	0.52	2.37
IRM7. Over 13 years old and sexually active	40.1	19.0	16.8	8.6	13.8	0.70	2.36
IRM8. Menstrual and/or gynaecological disorders	65.9	10.3	3.9	2.6	11.2	0.36	2.33
IRM9. Pregnancy tests	71.1	9.5	3.4	1.7	9.1	0.52	1.58
IRM10. Pregnancies/Abortions	82.3	2.2	0.9	0.9	7.3	0.35	1.39
IRM11. Sudden personality changes	50.4	22.4	15.1	7.3	0.9	0.68	2.69
IRM12. Important changes in physical appearance or way of dressing	76.7	7.8	5.6	2.2	2.2	0.57	1.03
Other Indicators of Risk (OIR)							1.03
OIR1. Minor is a sexual aggressor	80.6	0.9	1.3	1.3	10.3	0.10	1.03
OIR2. Emotional distress	19.0	28.9	30.6	19.8	1.3	0.73	1.03
OIR3. Signs and symptoms of depression	36.6	28.9	17.2	12.1	2.2	0.66	1.02
OIR4. Fears/State of alertness	47.8	24.1	12.5	6.5	5.2	0.65	1.02
OIR5. Anxiety	29.3	32.8	21.1	9.9	3.9	0.68	1.02
OIR6. Signs and symptoms of malnutrition	85.8	3.9	0.9	1.7	1.7	0.37	1.02
OIR7. Hypersexualised clothing	73.7	8.6	8.2	3.4	1.7	0.63	1.02
OIR8. Significant decrease in academic performance	65.5	12.5	5.6	8.2	3.4	0.61	1.02
OIR9. Irregular attendance at school	71.6	6.9	6.0	7.3	3.0	0.60	1.02
OIR10. Abuse of mobiles and social networks	44.0	19.0	15.5	11.2	6.9	0.54	2.49
OIR11. Sleeping problems	53.4	17.2	11.6	6.0	8.6	0.57	2.49
OIR12. Lack of social skills	39.7	28.0	17.2	8.2	3.0	0.37	2.49

**Table 3:** Percentage of participants in the different response options and factor loadings to vulnerabilities of CSE.

	Not Present	Present	Unknown		
	%	%	%	λ	VIF
Family Vulnerability Indicators (VF)					
VF1. Presence of any disability?	84.1	8.2	7.8	0.26	1.24
VF2. Migrant family or migrant minor	53.9	39.7	2.2	0.17	1.11
VF3. Inattention/Neglect/Abuse in childhood	10.8	82.8	5.6	0.48	1.10
VF4. Inappropriate living conditions	47.8	26.3	24.1	0.52	1.10
VF5. Family history of mental health issues	26.3	31.5	39.7	0.52	1.10
VF6. Family history of committing crimes	31.5	35.8	31.5	0.63	1.10
VF7. Family history of substance abuse	21.6	41.4	36.2	0.54	1.10
VF8. Family violence and/or gender-based violence inside the family	16.8	50.4	31.5	0.70	1.09
VF9. Family history of prostitution	47.0	6.5	45.7	0.64	1.09
VF10. Breakdown of family ties	21.1	73.3	4.7	0.40	1.09
Personal Vulnerability Indicators (VP)					1.17
VP1. Lack of positive ties with a protective adult	36.2	57.8	4.7	0.62	1.14
VP2. Prior history of bullying	48.3	17.7	32.8	0.61	1.14
VP3. Prior history of child sexual abuse	51.3	19.8	27.2	0.65	1.13
VP4. Sexualised behaviour of the minor	65.5	26.3	6.5	0.45	1.11
VP5. Eating disorders	79.7	10.8	7.8	0.37	1.11
VP6. Social isolation	44.8	22.8	4.7	0.52	1.10
VP7. Low self-esteem	28.9	65.1	5.6	0.56	1.24

**Table 4:** Descriptive statistics and mean differences in the total scores of the blocks of CSE according to sex and age group.

	Sex							Age Group							
	Min	Max	Mean	95% CI Mean		SD	Z_U_		Min	Max	Mean	95% CI Mean		SD	Z_U_
				LL	UL		(*p*)					LL	UL		(*p*)
CSE target indicators															
Male	0	15	2.26	1.63	2.89	3.03	4.68	11–13	0	18	1.89	0.60	3.17	3.74	15.0
Female	0	21	4.79	3.95	5.63	4.81	(<0.001)	14–17	0	21	4.13	3.47	4.79	4.51	(0.001)
Indicators of significant risk of CSE															
Male	0	16	4.36	3.47	5.25	4.31	0.08	11–13	0	10	2.51	1.53	3.50	2.87	8.47
Female	0	17	4.26	3.55	4.97	4.06	(0.933)	14–17	0	17	4.59	3.97	5.21	4.22	(0.014)
Indicators of moderate risk of CSE															
Male	0	15	3.43	2.75	4.12	3.30	4.88	11–13	0	15	1.89	0.86	2.91	2.99	31.41
Female	0	25	6.84	5.85	7.82	5.62	(<0.001)	14–17	0	25	6.12	5.37	6.88	5.14	(<0.001)
Other indicators of risk of CSE															
Male	0	24	6.23	5.12	7.34	5.36	3.46	11–13	0	24	7.63	5.43	9.82	6.39	2.76
Female	0	33	9.41	8.24	10.59	6.71	(0.001)	14–17	0	33	8.30	7.37	9.24	6.37	(0.251)
Vulnerabilities															
Male	1	12	5.67	5.12	6.23	2.67	3.50	11–13	0	14	6.40	5.28	7.52	3.26	0.20
Female	0	14	7.05	6.56	7.54	2.81	(<0.001)	14–17	1	14	6.49	6.08	6.90	2.79	(0.914)

## Data Availability

The data presented in this study are not publicly available due to ethical and confidentiality restrictions related to case-file information involving minors. The dataset consists of anonymized observations provided by professionals based on existing records. Data may be available from the corresponding author upon reasonable request and subject to ethical and institutional approval.
